# Association of *vitamin D receptor* gene rs739837 polymorphism with type 2 diabetes and gestational diabetes mellitus susceptibility: a systematic review and meta-analysis

**DOI:** 10.1186/s40001-022-00688-x

**Published:** 2022-05-07

**Authors:** Qiaoli Zeng, Dehua Zou, Yue Wei, Yingguang Ouyang, Zhaohang Lao, Runmin Guo

**Affiliations:** 1 Department of Internal Medicine, Shunde Women and Children’s Hospital (Maternity and Child Healthcare Hospital of Shunde Foshan), Guangdong Medical University, Foshan, 528300 Guangdong People’s Republic of China; 2 State Key Laboratory for Quality Research of Chinese Medicines, Macau University of Science and Technology, Taipa, Macau (SAR) People’s Republic of China; 3 Key Laboratory of Research in Maternal and Child Medicine and Birth Defects, Guangdong Medical University, Foshan, 528300 Guangdong People’s Republic of China; 4 Matenal and Child Research Institute, Shunde Women and Children’s Hospital (Maternity and Child Healthcare Hospital of Shunde Foshan), Guangdong Medical University, Foshan, 528300 Guangdong People’s Republic of China; 5 Department of Ultrasound, Shunde Women and Children’s Hospital (Maternity and Child Healthcare Hospital of Shunde Foshan), Guangdong Medical University, Foshan, 528300 Guangdong People’s Republic of China; 6 Department of General Affairs, Shunde Women and Children’s Hospital (Maternity and Child Healthcare Hospital of Shunde Foshan), Guangdong Medical University, Foshan, 528300 Guangdong People’s Republic of China; 7 Department of Endocrinology, Affiliated Hospital of Guangdong Medical University, Zhanjiang, 524001 Guangdong People’s Republic of China

**Keywords:** Type 2 diabetes mellitus, Gestational diabetes mellitus, *Vitamin D receptor*, rs739837, Susceptibility, Meta-analysis

## Abstract

**Background:**

Increasing evidence shows that genetic variants of genes in the diabetes mellitus (DM) metabolic pathway, such as *the vitamin D receptor* (*VDR*) gene rs739837 polymorphism, increase the risk of DM susceptibility. However, the findings have been inconsistent. The present study was performed to evaluate the association of *VDR* gene rs739837 and type 2 diabetes (T2DM) or gestational diabetes mellitus (GDM) risk.

**Methods:**

A comprehensive meta-analysis and a subgroup analysis were conducted to assess the association between *VDR* rs739837 and T2DM or GDM among five genetic models (dominant, recessive, homozygote heterozygote, and allele models) using a fixed or random model.

**Results:**

The meta-analysis included 9 studies. In the overall analysis, the results showed that *VDR* rs739837 was associated with an increased risk of T2DM or GDM in the allele model (T vs. G: *OR* = 1.088; 95% CI: 1.018–1.163; *P* = 0.012) and dominant model (TT + GT vs. GG: *OR* = 1.095; 95% CI: 1.001–1.197; *P* = 0.047). In the subgroup analysis, *VDR* rs739837 was also associated with an increased risk of T2DM in the allele model (T vs. G: *OR* = 1.159; 95% CI: 1.055–1.273; *P* = 0.002) and dominant model (TT + GT vs. GG: *OR* = 1.198; 95% CI: 1.048–1.370; *P* = 0.008). However, *VDR* rs739837 was not associated with GDM.

**Conclusions:**

Significant associations were found between the *VDR* rs739837 polymorphism and T2DM susceptibility, but not with GDM.

**Supplementary Information:**

The online version contains supplementary material available at 10.1186/s40001-022-00688-x.

## Introduction

Diabetes mellitus (DM) is a common chronic disorder that includes type 1 DM (T1DM), type 2 DM (T2DM), gestational DM (GDM) and other types of DM with T2DM accounting for the majority of the DM cases [1]. T2DM is a disease of multifactorial etiologies caused by insulin resistance and impaired insulin secretion [2]. Increasing evidence indicates that genetic factors contribute to T2DM susceptibility [3]. Moreover, genetic variations associated with insulin resistance and β-cell dysfunction have been suggested to play key roles in the development of GDM [4, 5]. Studies have shown that women with a history of GDM are at an increased risk of developing T2DM [6–8], and women with a family history of diabetes may have an increased risk of GDM [9]. Thus, GDM may share similar genetic susceptibilities and risk factors with T2DM [10–12].

Vitamin D (Vit D) has important immunoregulatory immune characteristics. Supplementation with vitamin D has been shown to prevent the development of T2DM [13]. Conversely, the depletion of vitamin D may be involved in the etiology of T2DM by influencing insulin secretion [14, 15]. Increasing evidence shows that genetic variants of genes in the DM metabolic pathway may increase the risk of DM susceptibility, such as *the vitamin D receptor* (*VDR*) gene, which is located on human chromosome 12q13.11 and is primarily expressed in the pancreas [16]. Alterations in *VDR* activation and expression may increase fatty acid synthase expression and lipogenesis [17, 18], resulting in a reduction in lipolysis and lipid deposition [19]. In addition, abnormal *VDR* expression contributes to calcium signal-mediated lipid accumulation through the p38 MAPK pathway [20], which results in high lipid deposition in the liver, disturbs insulin signaling and causes β-cell dysfunction [21]. Abnormal *VDR* expression may play an important role in the development of T2DM.

**Fig. 1:**
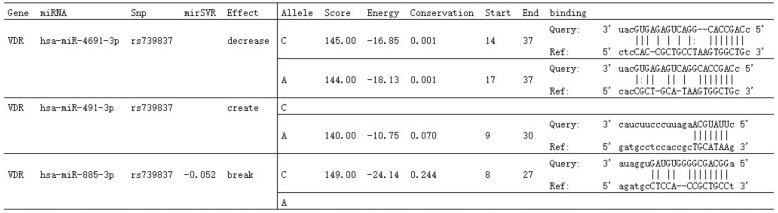
Effects of rs739837 on putative miRNA target sites

The rs739837 polymorphism is located in the 3′-untranslated region (UTR) of the *VDR* gene, and the rs739837 variation may affect *VDR* posttranscriptional regulation by binding with microRNA [22]. MiRNAs play an important role in the regulation of gene expression; thus, SNPs in the seed sites of miRNA targets may create or destroy miRNA-binding sites and further affect phenotypes and disease susceptibility [23]. We queried rs739837 polymorphism located in predicted miRNA target sites through the "MirSNP" database (http://bioinfo.bjmu.edu.cn/mirsnp/search/), which showed that rs739837 destroyed, created or disrupted putative miRNA target sites (Fig. 1). This polymorphism may affect the normal expression of *VDR* and further increase the risk of DM [24]. In recent years, several studies have shown associations of *VDR* rs739837 with T2DM [25–29] or GDM [30–32], but the results are controversial. Therefore, we conducted a meta-analysis to evaluate the association of *VDR* rs739837 with the risk of T2DM and GDM.

## Materials and methods

The present meta-analysis was conducted according to Preferred Reporting Items for Systematic Reviews and Meta-analyses (PRISMA) guidelines.

### Literature search

The Google Scholar, PubMed and Chinese National Knowledge Infrastructure databases were systematically searched for relevant studies using the following terms:

(1) “*VDR”* or “vitamin D receptor*”* or “rs739837” or “polymorphism” or “type 2 diabetes mellitus” and “T2DM”; and (2) “*VDR”* or “vitamin D receptor*”* or “rs739837” or “polymorphism” and “gestational diabetes mellitus” and “GDM”.

The search was performed with no date or language restrictions. All the studies were evaluated by reading the title and abstract to exclude irrelevant studies. The full texts of eligible studies were then assessed by reading the full text to confirm inclusion in the study.

### Inclusion and exclusion criteria

The inclusion criteria were as follows: (1) case–control/cohort studies; (2) studies that evaluated the association between *VDR* SNP rs739837 and T2DM/GDM; (3) adequate raw data or sufficient data to calculate odds ratios (ORs) with corresponding 95% confidence intervals (CIs); (4) a T2DM diagnosis based on the clinical criteria of the World Health Organization; and (5) a GDM diagnosis based on the clinical criteria of the World Health Organization.

The exclusion criteria were as follows: (1) not a case–control/cohort study; (2) not related to *VDR* SNP rs739837 and T2DM/GDM; (3) insufficient data; and (4) non-diabetic mellitus (NDM) subject data not in Hardy—Weinberg equilibrium (HWE).

### Data extraction

Two authors independently extracted the following data from the included studies:

first author; origin; year of publication; type of DM; numbers of T2DM/GDM patients and NDM controls; distribution of alleles and genotypes; and ORs with 95% CIs of the allele distribution**.**

### Statistical analysis

The following five genetic models were evaluated for rs739837: dominant model (TT + GT vs. GG), recessive model (TT vs. GG + GT), homozygote model (TT vs. GG), heterozygote model (GT vs. GG) and allele model (T vs. G). Genetic heterogeneity was estimated using the *Q*-test and I^2^ test. Lower heterogeneity was defined as *I*^2^ < 50% and *P* > 0.01 when using the fixed effects model (Mantel–Haenszel) to calculate ORs with corresponding 95% CIs. Otherwise, the random effects model (Mantel–Haenszel) was used [33, 34]. The significance of the ORs was evaluated using the Z test. Begg’s and Egger’s tests were used to determine publication bias. STATA v.14.0 software (Stata Corporation, TX, USA) was used to perform all statistical analyses.

## Results

### Study inclusion and characteristics

**Fig. 2:**
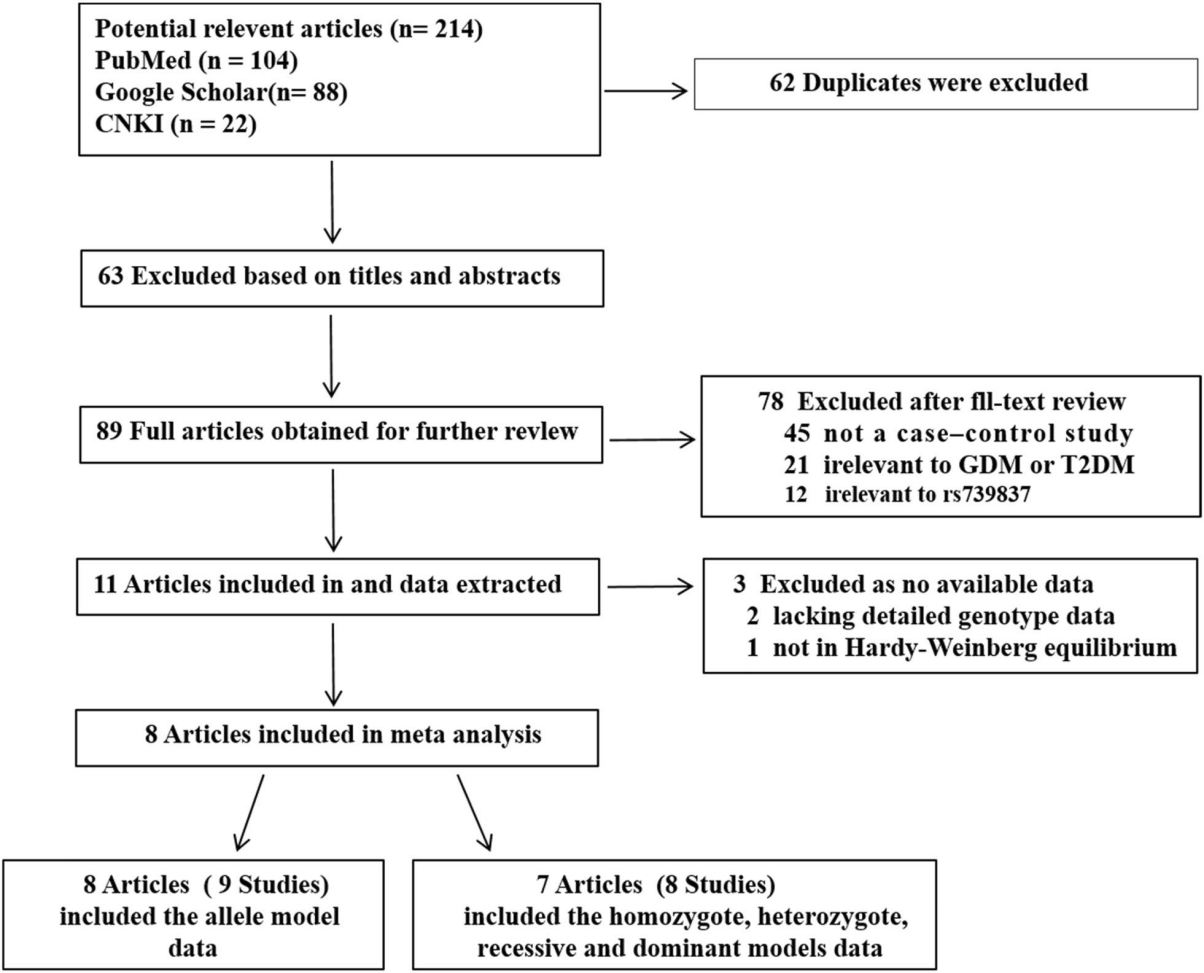
Flow diagram of study selection in the study

A total of 89 studies were searched using the inclusion and exclusion criteria. Figure 2 shows a flowchart of the study selection process. The following 9 eligible studies were included in the final analysis: 5 arteticles, which included 6 studies related to *VDR* SNP rs739837 and T2DM (one study only had allele mode data); and 3 articles, which included 3 studies related to *VDR* SNP rs739837 and GDM. The characteristics of each included study are shown in Table 1.

**Table 1:** Characteristics of each study included in this meta-analysis

Author	Year	Origin	Type	Cases/controls n	ORs with 95% CI (T vs G)	Allele distribution				Genotype distribution						
						Cases, n		Controls, n		Cases, n			Controls, n			
						G	T	G	T	GG	GT	TT	GG	GT	TT	HWE(*P*)
Zhang et al.	2021	Chinese (Henan)	T2DM	324/1687	1.238 (1.028–1.492)	459	189	2532	842	163	133	28	957	618	112	0.367
Yu et al.	2017	Chinese (Han)	T2DM	397/775	1.257(1.037–1.523)	565	229	1172	378	202	161	34	448	276	51	0.339
Lin et al.	2016	Chinese (Neimenggu)	T2DM	319/387	0.987 (0.779–1.251)	469	169	567	207	171	127	21	209	149	29	0.699
Vimaleswaran et al.	2014	British	T2DM	–	1.162 (0.937–1.441)	–	–	–	–	–	–	–	–	–	–	> 0.57
Xu et al.	2014	Chinese (Ningxia Hui population)	T2DM	154/115	1.089 (0.752–1.577)	210	98	161	69	69	72	13	54	53	8	> 0.05
Xu et al.	2014	Chinese (Ningxia Han population)	T2DM	201/148	1.066 (0.778–1.461)	259	143	195	101	93	73	35	70	55	23	> 0.05
Chen et al.	2021	Chinese (Guangdong)	GDM	555/646	0.953 (0.801–1.134)	776	334	890	402	281	214	60	313	264	69	0.236
Liu et al.	2021	Chinese (Hubei)	GDM	816/851	1.058 (0.910–1.229)	1152	480	1221	481	414	324	78	447	327	77	> 0.05
Wang et al.	2015	Chinese (Beijing)	GDM	657/772	1.047 (0.889–1.232)	935	379	1113	431	334	267	56	401	311	60	0.874

*n* number, *T2DM* type 2 diabetes mellitus, *GDM* gestational diabetes mellitus, *OR* odds ratio, *CI* confidence interval, *HWE* Hardy–Weinberg equilibrium, (-) not applicable

### Heterogeneity analysis

#### Overall heterogeneity analysis

**Fig. 3:**
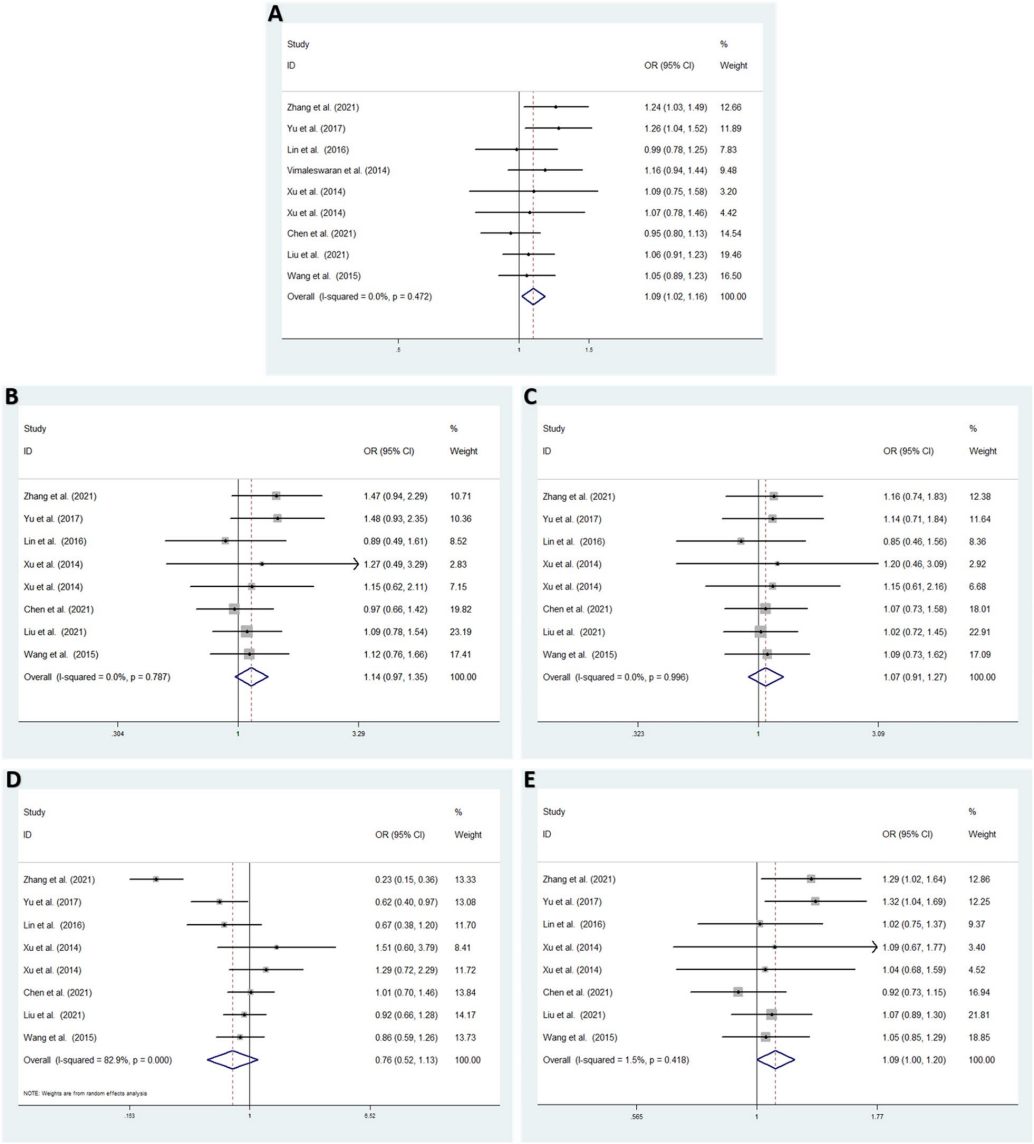
The overall meta-analysis for the association between VDR rs739837 and T2DM or GDM susceptibility. **A** Allele model: T vs. G (fixed effects model). **B** Homozygote model: TT vs. GG (fixed effects model). **C** Heterozygote model: GT vs. GG (fixed effects model). **D** Recessive model: TT vs. GG + GT (random effects model). **E** Dominant model, TT + GT vs. GG (fixed effects model). *OR* odds ratio, *CI* confidence interval, I2: measurement to quantify the degree of heterogeneity in meta-analyses

Low heterogeneity among studies was detected in the allele model (T vs. G: *I*^2^ = 0.0%; *P* = 0.472) [25–32], homozygote model (TT vs. GG: *I*^2^ = 0.0%; *P* = 0.787), heterozygote model (GT vs. GG: *I*^2^ = 0.0%; *P* = 0.996) and dominant model (TT + GT vs. GG: *I*^2^ = 1.5%; *P* = 0.418). High heterogeneity was detected in the recessive model (TT vs. GG + GT: *I*^2^ = 82.9%; *P* < 0.001) [25, 26, 28–32] (Fig. 3).

#### Subgroup heterogeneity analysis

**Fig. 4:**
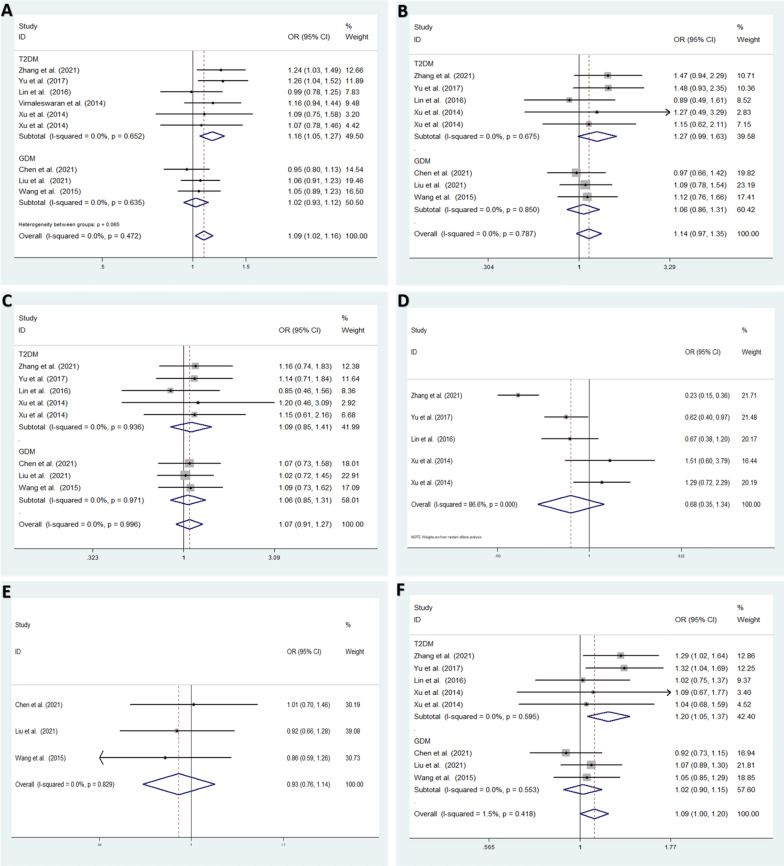
Subgroup meta-analysis for the association between VDR rs739837 and T2DM or GDM susceptibility. **A** Allele model: T vs. G (fixed effects model). **B** Homozygote model: TT vs. GG (fixed effects model). **C** Heterozygote model: GT vs. GG (fixed effects model). **D** Recessive model (T2DM): TT vs. GG + GT (random effects model). **E** Recessive model (GDM): TT vs. GG + GT (fixed effects model). **F** Dominant model, TT + GT vs. GG (fixed effects model). *OR* odds ratio, *CI* confidence interval, I2: measurement to quantify the degree of heterogeneity in meta-analyses

In the T2DM subgroup, low heterogeneity among studies was detected in the allele model (T vs. G: *I*^2^ = 0.0%; *P* = 0.652) [25–29], homozygote model (TT vs. GG: *I*^2^ = 0.0%; *P* = 0.675), heterozygote model (GT vs. GG: *I*^2^ = 0.0%; *P* = 0.936) and dominant model (TT + GT vs. GG: *I*^2^ = 0.0%; *P* = 0.595). High heterogeneity was detected in the recessive model (TT vs. GG + GT: *I*^2^ = 86.6%; *P* < 0.001) [25, 26, 28, 29] (Fig. 4).

In the GDM subgroup, low heterogeneity among studies was detected in the allele model (T vs. G: *I*^2^ = 0.0%; *P* = 0.635), homozygote model (TT vs. GG: *I*^2^ = 0.0%; *P* = 0.850), heterozygote model (GT vs. GG: *I*^2^ = 0.0%; *P* = 0.971), recessive model (TT vs. GG + GT: *I*^2^ = 0.0%; *P* = 0.829) and dominant model (TT + GT vs. GG: *I*^2^ = 0.0%; *P* = 0.553) [30–32] (Fig. 4).

### Overall meta-analysis results

In the overall analysis, a fixed effects model was used to analyze the allele, homozygote, heterozygote and dominant models. *VDR* rs739837 was shown to be significantly associated with increased DM (T2DM and GDM) risk in the allele model (T vs. G: *OR* = 1.088; 95% CI: 1.018–1.163; *P* = 0.012) and dominant model (TT + GT vs. GG: *OR* = 1.095; 95% CI: 1.001–1.197; *P* = 0.047). No significant associations were found under the homozygote model (TT vs. GG: *OR* = 1.144; 95% CI: 0.973–1.346; *P* = 0.103) and heterozygote model (GT vs. GG: *OR *= 1.073; 95% CI: 0.909–1.266; *P* = 0.406). A random effects model indicated no significant difference for the recessive model (TT vs. GG + GT: *OR* = 0.764; 95% CI: 0.517–1.129; *P* = 0.177) (Fig. 3).

### Subgroup meta-analysis results

We performed subgroup analysis according to the type of DM to evaluate the association between *VDR* rs739837 and T2DM or GDM susceptibility.

In the T2DM subgroup, the results showed that rs739837 was significantly related to an increased risk of T2DM in the allele model (T vs. G: *OR* = 1.159; 95% CI: 1.055–1.273; *P* = 0.002) and dominant model (TT + GT vs. GG: *OR *= 1.198; 95% CI: 1.048–1.370; *P* = 0.008) using a fixed effects model. No significant associations were found under the homozygote model (TT vs. GG: *OR* = 1.273; 95% CI: 0.992–1.633; *P* = 0.058) or heterozygote model (GT vs. GG: *OR* = 1.094; 95% CI: 0.849–1.410; *P* = 0.486) using a fixed effects model. A random effects model also showed that no significant difference was found for the recessive model (TT vs. GG + GT: *OR* = 0.269; 95% CI: 0.684–0.349; *P* = 0.269) (Fig. 4).

In the GDM subgroup, no significant associations were found under the allele model (T vs. G: *OR* = 1.023; 95% CI: 0.932–1.123; *P* = 0.631), homozygote model (TT vs. GG: *OR* = 1.060; 95% CI: 0.857–1.313; *P* = 0.590), heterozygote model (GT vs. GG: *OR* = 1.057; 95% CI: 0.850–1.315; *P* = 0.618), recessive model (TT vs. GG + GT: *OR* = 0.931; 95% CI: 0.758–1.143; *P* = 0.493) or dominant model (TT + GT vs. GG: *OR* = 1.018; 95% CI: 0.903–1.148; *P* = 0.765) using a fixed effects model (Fig. 4).

### Publication bias

**Fig. 5:**
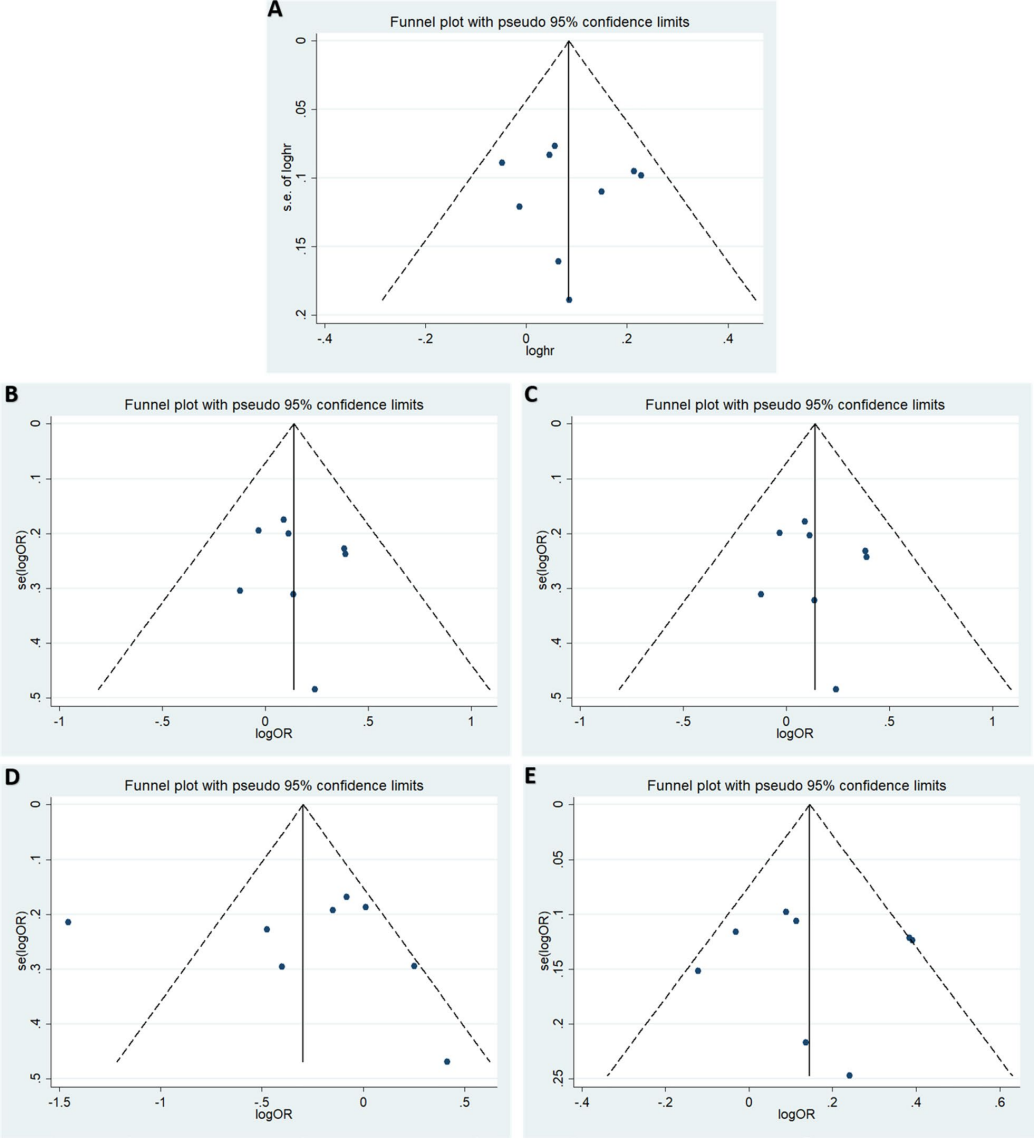
Funnel plot of the odds ratios in the overall meta-analysis. **A** Allele model: T vs. G. **B** Homozygote model: TT vs. GG. **C** Heterozygote model: GT vs. GG. **D** Recessive model, TT vs. GG + GT. **E** Dominant model: TT + GT vs. GG

**Fig. 6:**
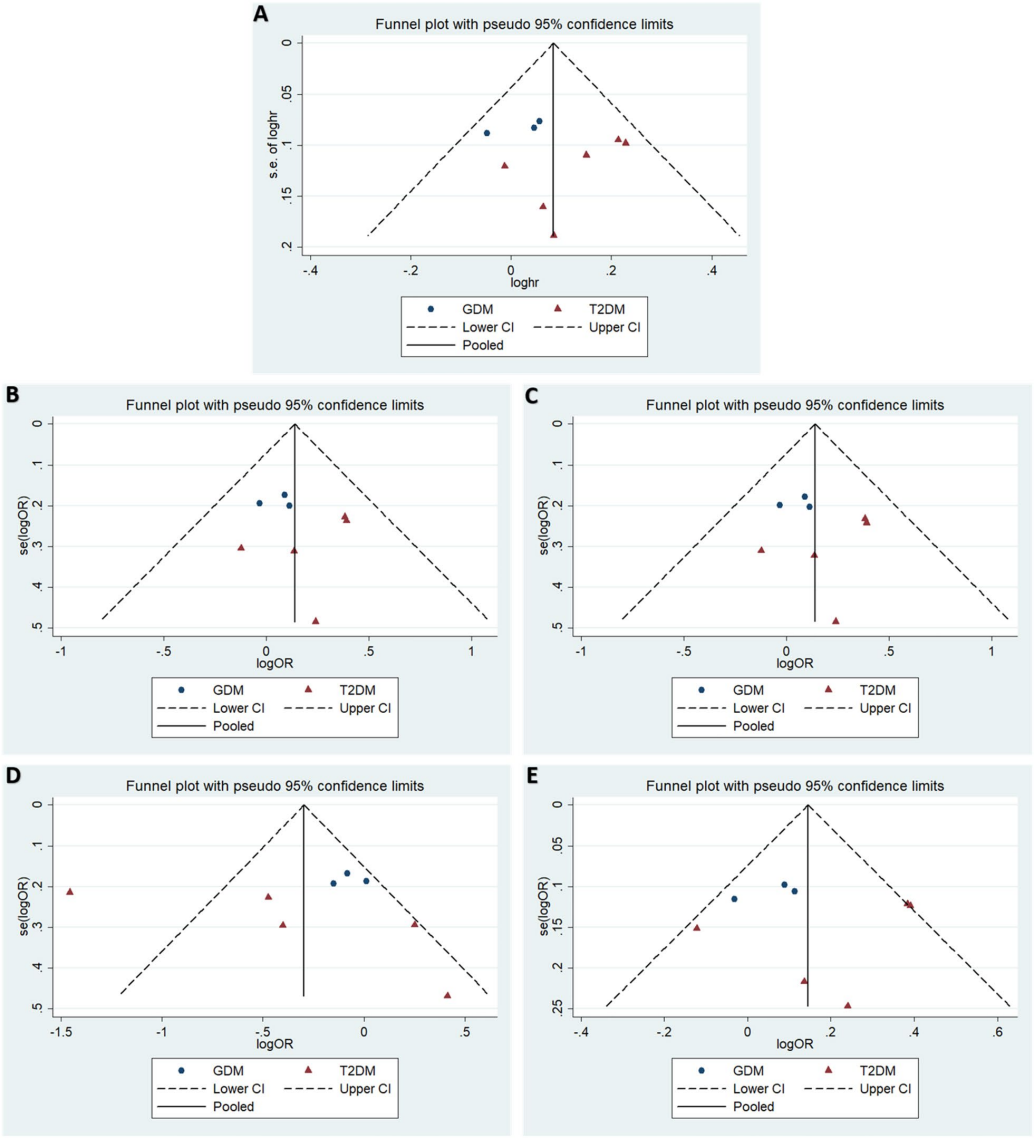
Funnel plot of the odds ratios in the subgroup meta-analysis. **A** Allele model: T vs. G. **B** Homozygote model: TT vs. GG. **C** Heterozygote model: GT vs. GG. **D** Recessive model, TT vs. GG + GT. **E** Dominant model: TT + GT vs. GG

According to Begg’s and Egger’s tests, no significant publication bias was found in any of the genetic models (all *P* > 0.05, Additional file 1: Tables S1–S3), and the funnel plots are shown in Figs. 5, 6.

## Discussion

In this systematic review and meta-analysis study, we performed a systematic and objective assessment of the associations between the *VDR* rs739837 polymorphism and DM. The findings of the meta-analysis of 9 case–control studies in the overall type of DM determined a significant association of the T allele and TT + GT genotype with DM risk. Moreover, the subgroup analysis also revealed that rs739837 was significantly related to an increased risk of T2DM in the T allele and TT + GT genotype, but no significant associations were found under any models in the GDM subgroup.

The *VDR* gene has been confirmed to be significantly involved in the regulation of the endocrine system, suggesting that it is a potential candidate gene for metabolic disorders. Rs739837 is located in the 3′-untranslated region (UTR) of the *VDR* gene, which regulates gene expression. A series of investigations have reported that the rs739837 SNP is associated with diabetes risk. Zhang et al. demonstrated a significant association between the T allele and TT + GT genotype of rs739837 and T2DM risk [25]. Yu et al. found a significant relationship between the rs739837 polymorphism and T2DM in the T allele, recessive model (GG/GT + TT) and additive model (GG/TT) [26]. A previous study has identified that the rs739837 genotype distributions show significant differences across T2DM cases and controls [28]. Interestingly, Jia et al. found that the T and C allele frequencies of rs739837 are 70.2% and 29.8% in cases, respectively, and 73 and 27%, in controls, respectively. The control group results reported by Jia et al. were inconsistent with other reports; they reported that rs739837 is significantly associated with an increased risk of T2DM in the additive model (TT vs. TC vs. CC) and dominant (TT vs. TC/CC) model [35]. Due to the differences in allele frequencies from other reports, the study by Jia et al. was not included in the meta-analysis. Vimaleswaran and Lin showed that rs739837 is not associated with T2DM risk [27, 29]. Moreover, four studies demonstrated no relationship between the genotypic model of rs739837 and GD [30–32, 36]. A previous meta-analysis has suggested that women with a history of GDM are almost 10 times more likely to develop T2DM than those with a normoglycemic pregnancy [7]. The magnitude of this risk is consistent with evidence that T2DM and GDM share common pathogenic mechanisms and risk factors.

Interestingly, in the overall analysis, low heterogeneity among studies was detected in the allele, dominant, homozygote and heterozygote genetic models, and high heterogeneity among studies was detected under the recessive model as well as in the subgroup analysis. Although the GDM studies only contained women, there was no significant heterogeneity in the meta-analysis with T2DM research involving men and women, except for the recessive model. The subtype analysis revealed that the T2DM group had higher heterogeneity, while the GDM group had lower heterogeneity. Therefore, it was possible to combine T2DM and GDM for meta-analysis to explore potential common susceptibility factors. However, due to the small GDM sample size, there was no powerful conclusion of the results.

There were several limitations in the present meta-analysis. First, there were limited studies that estimated *VDR* rs739837 and T2DM or GDM risk. In particular, few articles have researched the association between *VDR* rs739837 and GDM. Only three articles contained data from five genetic models, and one article did not have available data for meta-analysis, which may affect the overall estimation. Moreover, the present study included only Chinese studies. Thus, studies using larger sample sizes of other ethnic groups worldwide need to be performed. Finally, the present study only evaluated the association between rs739837 genotypes and T2DM or GDM risk without adjusting the effects of other risk factors, such as interacting gene–gene and gene–environment factors [37]. Therefore, further study is required to evaluate the susceptibility factors of T2DM or GDM.

## Conclusions

To our knowledge, this study is the first to assess the role of *VDR* rs739837 and T2DM or GDM risk. Significant associations were found between the *VDR* rs739837 polymorphism and T2DM susceptibility but not association with GDM.

## Supplementary Information

**Additional file 1: Table S1** Begg’s and Egger’s tests in overall analysis. **Table S2** Begg’s and Egger’s tests in T2DM subgroup analysis. **Table S3** Begg’s and Egger’s tests in GDM subgroup analysis.

## Data Availability

All data used or generated during the study are available from the corresponding author on reasonable request: Runmin Guo, E-mail: 1314ivu@126.com.
