# Supplementary material for: Association of vitamin D receptor gene rs739837 polymorphism with type 2 diabetes and gestational diabetes mellitus susceptibility: a systematic review and meta-analysis
Source: Eur J Med Res. 2022 May 7;27:65. doi: 10.1186/s40001-022-00688-x (PMC9080160; doi:10.1186/s40001-022-00688-x)
Supplement: Supplementary file 1 [file 40001_2022_688_MOESM1_ESM.docx]

Table S1: Begg's and Egger's tests in overall analysis.

Genetic models	Begg's Test (*P*)	Egger's test (*P*)
T vs. G	0.754	0.839
TT vs. GG	0.386	0.767
GT vs. GG	0.386	0.769
TT vs. GG + GT	0.536	0.804
TT +GT vs. GG	0.536	0.921

Table S2: Begg's and Egger's tests in T2DM subgroup analysis.

Genetic models	Begg's Test (*P*)	Egger's test (*P*)
T vs. G	0.188	0.189
TT vs. GG	0.327	0.412
GT vs. GG	0.327	0.400
TT vs. GG + GT	0.142	0.161
TT +GT vs. GG	0.624	0.502

Table S3: Begg's and Egger's tests in GDM subgroup analysis.

Genetic models	Begg's Test (*P*)	Egger's test (*P*)
T vs. G	0.117	0.318
TT vs. GG	0.602	0.857
GT vs. GG	0.602	0.845
TT vs. GG + GT	0.602	0.938
TT +GT vs. GG	0.602	0.429
